# Sequential Isolation and Characterization of Single CTCs and Large CTC Clusters in Metastatic Colorectal Cancer Patients

**DOI:** 10.3390/cancers13246362

**Published:** 2021-12-18

**Authors:** Federica Francescangeli, Valentina Magri, Maria Laura De Angelis, Gianluigi De Renzi, Orietta Gandini, Ann Zeuner, Paola Gazzaniga, Chiara Nicolazzo

**Affiliations:** 1 Department of Oncology and Molecular Medicine, Istituto Superiore di Sanità, Viale Regina Elena 299, 00161 Rome, Italy; federica.francescangeli@iss.it (F.F.); marialaura.deangelis@iss.it (M.L.D.A.); a.zeuner@iss.it (A.Z.); 2 Department of Radiology, Oncology and Pathology, Sapienza University of Rome, Viale Regina Elena 324, 00161 Rome, Italy; valentina.magri@uniroma1.it; 3 Cancer Liquid Biopsy Unit, Department of Molecular Medicine, Sapienza University of Rome, 00161 Rome, Italy; gianluigi.derenzi@uniroma1.it (G.D.R.); orietta.gandini@uniroma1.it (O.G.); paola.gazzaniga@uniroma1.it (P.G.)

**Keywords:** circulating tumor cells, CTC cluster, colorectal cancer, size-based method, ScreenCell^®^, epithelial mesenchymal transition, hypoxia, HIF-1α, immunofluorescence analysis, sequential filtration

## Abstract

**Simple Summary:**

The presence of cancer cells clusters is a frequent event capable of increasing their aptitude to survive in the bloodstream. Consistently, clusters ranging from 2–50 cancer cells are detected in about 50% of patients with metastatic cancers, including colorectal carcinoma. Although a deepened analysis of clusters might certainly offer new insights into the complexity of metastatic cascade, research in this field has come to a halt, since most circulating tumor cells isolation techniques are not compatible with large-sized clusters isolation. In the present study, we describe a sequential method to simultaneously isolate single and clustered circulating tumor cells from a single blood draw, opening new scenarios for an ever more precise characterization of colorectal cancer metastatic cascade.

**Abstract:**

Circulating tumor cells (CTCs) detach from a primary tumor or its metastases and circulate in the bloodstream. The vast majority of CTCs are deemed to die into the bloodstream, with only few cells representing viable metastatic precursors. Particularly, single epithelial CTCs do not survive long in the circulation due to the loss of adhesion-dependent survival signals. In metastatic colorectal cancer, the generation of large CTC clusters is a very frequent occurrence, able to increase the aptitude of CTCs to survive in the bloodstream. Although a deepened analysis of large-sized CTC clusters might certainly offer new insights into the complexity of the metastatic cascade, most CTC isolation techniques are unfortunately not compatible with large-sized CTC clusters isolation. The inappropriateness of standard CTC isolation devices for large clusters isolation and the scarce availability of detection methods able to specifically isolate and characterize both single CTCs and CTC clusters finally prevented in-depth studies on the prognostic and predictive value of clusters in clinical practice, unlike that which has been described for single CTCs. In the present study, we validated a new sequential filtration method for the simultaneous isolation of large CTC clusters and single CTCs in patients with metastatic colorectal cancer at failure of first-line treatments. The new method might allow differential downstream analyses for single and clustered CTCs starting from a single blood draw, opening new scenarios for an ever more precise characterization of colorectal cancer metastatic cascade.

## 1. Introduction

Circulating tumor cells (CTCs) detach from a primary tumor or its metastases and circulate in the bloodstream [1]. Beyond the unquestionable prognostic value of the number of CTCs in patients with metastatic solid tumors, a detailed molecular characterization of CTCs is critical to improve our understanding of key pathways that mediate the dissemination of cancer cells [2,3]. The vast majority of CTCs are deemed to die into the bloodstream, with only few cells representing viable metastatic precursors [4]. Particularly, single epithelial CTCs do not survive long in the circulation due to the loss of adhesion-dependent survival signals [5]. Therefore, the interaction with other CTCs generating CTC clusters has been described as a frequent event able to increase their aptitude to survive in the bloodstream [6]. Consistently, clusters of CTCs ranging from 2–50 cancer cells are detected in about 50% of patients with metastatic cancers, and are associated with worse prognosis [7]. In colorectal cancer, the presence of CTC clusters has been widely described and correlated with elevated circulating levels of transforming growth factor-β (TGF-β) [8]. Recent studies suggest that CTC clusters and single CTCs display distinct gene expression profiles and molecular features, which might account for their different metastatic propensity [2]. Transcriptome analyses have shown that CTC clusters often display mixed epithelial and mesenchymal features and that proteins involved in desmosome junctions, such as plakoglobin, are preferentially expressed in clusters compared to single cells [9]. Moreover, some evidence has been provided that large CTC clusters are protected from reactive oxygen species by activating the metabolic switch to glycolysis through hypoxia-inducible factor-1α (HIF-1α) [10]. Although a deepened analysis of CTC clusters might certainly offer new insights into the complexity of the metastatic cascade, research in this field has come to a halt, since most CTC isolation techniques are not compatible with large-sized CTC clusters isolation. In this regard, despite the large body of evidence that has been provided showing that CTC clusters are usually enriched in mesenchymal markers, the clinical significance of CTC clusters has been mostly demonstrated using the CellSearch^®^ (Menarini Silicon Biosystems, Castel Maggiore, BO, Italy), an antigen-dependent method able to isolate only clusters with epithelial features, missing CTC clusters undergoing epithelial–mesenchymal transition (EMT) [11]. Apart from EMT, other explanations, such as clusters’ disruption in devices with turbulent flow, might account for their underestimation when using antigen-dependent assays. The failure of antigen-dependent methods to capture CTC clusters paved the way for alternative antigen-independent methods for CTC isolation [12]. Among them, ScreenCell^®^ (ScreenCell, Sarcelles, France) is a filtration method allowing the isolation of CTCs by size using a filter with 6.5 to 8 µm pores. The rationale is that CTCs are generally larger in size than hematopoietic cells, so most of these cells pass through the filter whereas CTCs and clusters are retained [13]. Different downstream analyses such as immunocytochemistry, immunofluorescence, DNA or RNA extraction for genomic study can be directly performed on the filter in order to characterize CTCs [14]. However, in order to perform differential downstream analyses for single CTCs and CTC clusters, a single filter is not sufficient, making it necessary for this purpose to increase the starting blood volume to obtain more filters. In the present study we described a new method for the simultaneous isolation of CTC clusters and single CTCs from a single blood draw through a sequential filtration, using adapted ScreenCell^®^ filters with increased pore size. We validated the assay in a small population of patients with metastatic colorectal cancer at failure of first line treatments.

## 2. Materials and Methods

### 2.1. Blood Samples Collection

Ten patients with metastatic colorectal cancer at failure of first-line treatments have been enrolled. For each patient, peripheral blood was collected into a K_2_EDTA tube, stored at +4 °C and processed within 3 h after drawing. Informed consent was obtained from all participants included in the study. The protocol was approved by Ethical Committee of Policlinico Umberto I (protocol n. 668/09, 9 July 2009; amended protocol 179/16, 1 March 2016). Characteristics of CTC-positive patient population are shown in Table 1.

### 2.2. Establishment of a Customized Filtration Method for the Isolation of CTCs Clusters

ScreenCell^®^ (ScreenCell, Sarcelles, France) is a simple, non-invasive technology for isolating CTCs from whole blood. The ScreenCell^®^ filtration devices were developed in order to isolate CTCs by size on a microporous membrane filter. These devices are designed for isolation of: (a) fixed cells for cytological studies (ScreenCell^®^ Cyto); (b) live cells for culture (ScreenCell^®^ CC) and (c) molecular biology (ScreenCell^®^ MB) [14]. The filter allows fast and regular filtration, preserving the CTCs morphology and structures. At the end of filtration, the ScreenCell^®^ Cyto filter is released onto a standard microscopy glass slide. Cytological studies including staining, cell enumeration, immunocytochemistry and FISH assays, can then be conducted directly on the filter. The circular filter of the ScreenCell^®^ device is composed of polycarbonate material, with a smooth flat and hydrophilic surface. Circular pores are calibrated for isolation of fixed or live cells and randomly distributed throughout the filter (1 × 10^5^ pores/cm^2^). In order to enable the selective filtration of large CTC clusters we aimed to modify the size of the pores, increasing it to 15 µm size. These adapted devices were referred as ScreenCell Cyto-Cl.

### 2.3. Sequential Isolation of Single CTCs and of CTC Clusters

In order to simultaneously isolate single CTCs and CTC clusters, sequential filtration was performed first using the new ScreenCell Cyto-Cl kit specifically designed and adapted to isolate cell clusters and then the ScreenCell^®^ Cyto kit to isolate single cells. Blood samples were collected using tubes containing K_2_EDTA, stored at +4 °C and processed within 3 h. Briefly, 3 mL of blood was diluted in 4 mL of fixed cells (FC2) dilution buffer allowing lysis of red blood cells (RBC) while preserving other cells. After 8 min of incubation at room temperature, 7 mL of diluted sample was put into device tank of ScreenCell Cyto-Cl device and filtered under a pressure gradient created by a vacutainer tube. After washing with phosphate-buffered saline (PBS) to remove RBC debris, the filter was left on absorbing paper to dry at room temperature. Thereafter, all clusters-depleted blood samples were filtrated using ScreenCell^®^ Cyto device to isolate residual single CTCs. After washing with PBS, the filter was left on absorbing paper to dry at room temperature. Filters were stored at −20 °C until downstream analysis. Each filtration was usually completed within 3 min.

### 2.4. Immunofluorescence Staining

For immunofluorescence, filters were hydrated with Tris-Buffered Saline (TBS) for 10 min and directly stained with antihuman biotinylated CD45 (#130-098-551, Miltenyi Biotec, Bologna, Italy) in order to eliminate hematopoietic cells as follows: filters were washed twice in TBS 0.002% Tween20, endogenous peroxidase activities were blocked using 0.03% hydrogen peroxide for 15 min in the dark, then the sections were incubated at room temperature for 1 h 30 min with CD45 biotinylated antibody. Sections were then processed using streptavidin conjugated to horseradish peroxidase and substrate–chromogen solution both contained in UltraTek HRP Anti-Polyvalent DAB kit (#AMF080, Scytek Laboratories, Logan, UT, USA), following the manufacturer’s instructions. Samples were then incubated in a humid chamber overnight at +4 °C with the following primary antibodies goat antihuman CK20 (#SC-17113, Santa Cruz Biotechnology, Dallas, TX, USA), rabbit antihuman HIF-1α (#36169, Cell Signaling Technology, Danvers, MA, USA) and mouse antihuman Vimentin (#SC-373717, Santa Cruz Biotechnology) in a humid chamber overnight at +4 °C. The filters were then washed twice in PBS and incubated with a mixture of appropriate secondary antibodies: donkey anti-mouse IgG Alexa Fluor^®^488-conjugated (#A21202), donkey anti-goat IgG Alexa Fluor^®^647-conjugated (#A21447) and donkey anti-rabbit IgG Alexa Fluor^®^555-conjugated (#A31572) for 45 min at room temperature in the dark. Nuclei were stained with DAPI for 15 min at room temperature. All antibodies were dissolved in PBS containing 3% bovine serum albumin (BSA), 3% fetal bovine serum (FBS), 0.001% NaN_3_ and 0.1% Triton X-100. Finally, the filters were mounted with Prolong-Gold Antifade (Thermo Fisher Scientific, Waltham, MA, USA) on slides and analyzed using a Zeiss LSM900 confocal microscope or an Olympus FV1000 confocal microscope equipped with 60× oil immersion objectives. 

## 3. Results

We sought to investigate the efficacy of the double filtration system in isolating all circulating tumor cells, including large clusters, regardless of surface markers. All blood samples were successfully filtered. In seven blood samples we were able to detect 42 single CTCs, with a range in number from 3 to 9 per 3 mL of blood, and 31 CTC clusters with large dimension, with a range 3 to 6 per 3 mL of blood, as shown in Table 2. Interestingly, both CTC and CTC clusters were detected in these patients. Conversely, in three patients we were not able to detect CTCs or CTCs clusters.

**Figure 1:**
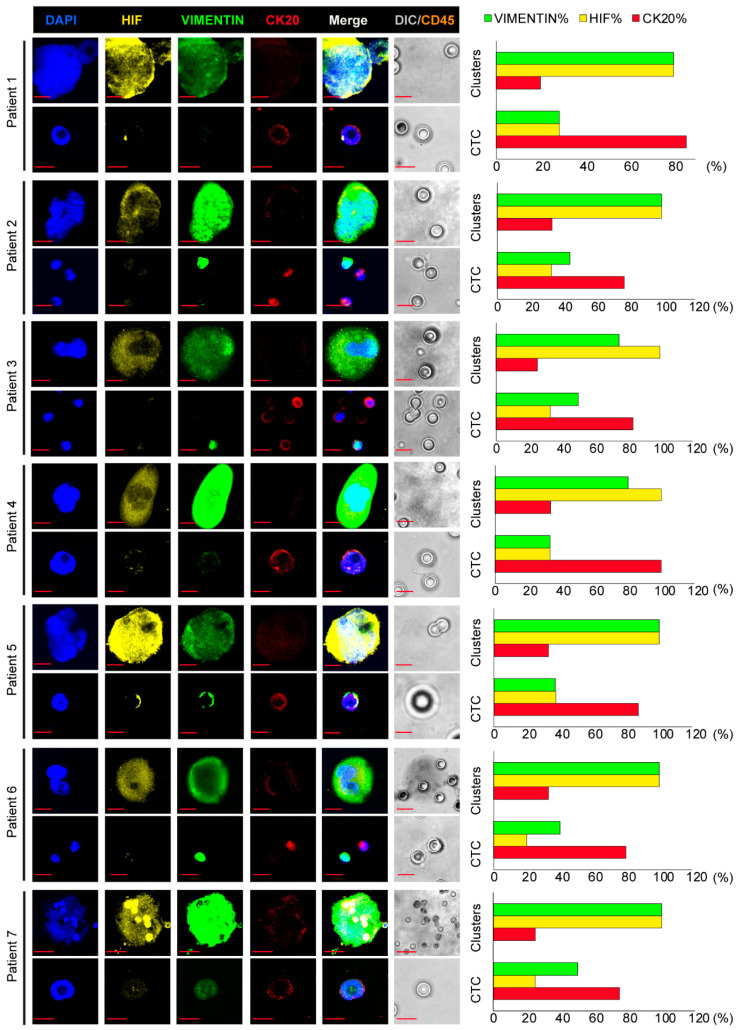
Illustrative images of triple immunofluorescence assay on clusters and circulating colon cancer cells. (**Left**) Representative confocal images of CTC clusters and CTCs stained with anti-CK20 (**red**), anti-vimentin (**green**) and anti-HIF-1α (**yellow**) antibodies. (**Right**) Graphical representation of percent of single or clustered CTCs expressing CK20 (**red bars**), vimentin (**green bars**), HIF-1α (**yellow bars**). Magnification 60×, 5× zoom bar 10 µm. CTC: circulating tumor cell; CK: cytokeratin; HIF: hypoxia-inducible factor.

Hypoxia and EMT-like features were investigated in order to assess whether EMT was associated with HIF-1α in both single and clustered CTCs. For this purpose, a triple immunofluorescence staining for CK20, vimentin and HIF-1α was carried out (Figure 1). Hematopoietic cells were preventively excluded by staining each filter for CD45, as shown in Figure S1. According to our hypothesis, CK20 was predominantly expressed in single CTCs. In fact, the antigen was found expressed in 83.3% out of the 42 single CTCs analyzed, whilst it was found expressed in 29% out of the 31 CTC clusters (Table 2). Conversely, vimentin and HIF-1α were mostly detected in CTC clusters. Indeed, HIF-1α and vimentin were found expressed in 84 and 77% out of the 31 CTC clusters, respectively; whilst HIF-1α was detected in 31% out of the 42 single CTCs and vimentin in 40.5% (Table 2). 

Clusters showed a different phenotype compared to CTCs, by reflecting hybrid-EMT features, with a poor or barely detectable CK20 expression (Figure 1left and Figure S2) in a percentage ranging from 20 to 33 as shown by graphs on the right (red bar); while Vimentin and HIF increased their expression ranging from 75 to 100 (%) and from 80 to 100 (%), respectively, as shown in images on the left of Figure 1. These data suggest a role for EMT and HIF-1α in large cluster organization. Altogether, these observations indicate that this filtration system is valid and effective, allowing collection and analysis even of large clusters that would have been excluded by epithelial antibodies-based methods for CTC detection.

## 4. Discussion

Circulating tumor cells clusters represent a peculiar class of CTCs, with specific properties including reduced apoptosis, enhanced survival and high metastatic potential. Unlike single CTCs, CTC clusters have not been deeply investigated, mainly due to the paucity of reliable detection methods [15]. In fact, most assays for CTC clusters enrichment, which depend on epithelial specific markers such as cytokeratins (CKs), and the epithelial cell adhesion molecule (EpCAM), usually underestimate CTC clusters due to their hybrid epithelial–mesenchymal features. A further limitation of antibody-based methods, such as the FDA-approved CellSearch^®^, is that larger CTC clusters have a small area-to-volume ratio, thus preventing correct binding to the antibodies used in the enrichment step [16]. The inappropriateness of standard CTCs isolation devices for clusters isolation and the scarce availability of detection methods able to specifically isolate and characterize both single CTCs and CTC clusters finally banned in-depth studies on the prognostic and predictive value of clusters in clinical practice, unlike that which has been described for single CTCs. Although evidence has been provided that size-exclusion assays, such as blood filtration, would represent an affordable approach for CTC clusters isolation, only few filtration devices can simultaneously detect single and clustered CTCs starting from a single blood sample [17]. Here we used a sequential filtration-based approach to investigate the simultaneous presence of single CTCs and CTC clusters in a small group of patients with metastatic colorectal cancer. The ScreenCell^®^ technology, which we had previously used for single CTCs isolation and characterization in non-small cell lung cancer (NSCLC) and colorectal cancer [18,19], was adapted for CTC clusters isolation by increasing the filter pore size. This innovative double-step filtration allows a rapid, easy and simultaneous enrichment of both single and clustered CTCs from a single blood draw, obtaining two filters that can be easily subjected to specific downstream analyses. In this pilot study we sought to investigate the efficacy of the double filtration method to simultaneously isolate single and clustered CTCs, and to clarify whether they might display distinct molecular features, mainly in terms of hybrid EMT-related characteristics. The double-filtration method described herein allowed us to isolate in all patients both single CTCs and large clusters, confirming what we and others have previously demonstrated, namely that in colon carcinoma the presence of large clusters is a very frequent phenomenon, usually associated with higher levels of TGF-β in circulation [20,21]. Consistently with literature studies, CTC clusters display manifest hybrid-EMT features compared to single CTCs, as demonstrated by the constant expression in CTC clusters of vimentin and CK20, with vimentin always expressed to a much higher extent. The choice to include HIF-1α in the triple immunofluorescence experiments was dictated by the unequivocal role that hypoxia plays in the generation of CTC clusters [22]. In fact, recent studies have demonstrated that hypoxic cancer cells are characterized by upregulated cell–cell junction components, a property that seems to be associated with their propensity to frequently intravasate as clusters rather than as individual CTCs [23]. Our results confirm that, in all the patients analyzed, single CTCs significantly differ from clusters in terms of HIF-1α ex pression, HIF-1α being constantly expressed in large clusters, but not in single CTCs.

## 5. Conclusions

This liquid biopsy test seems promising for the future isolation and characterization of different CTCs subtypes, including clusters. The advantages of this test compared to others currently in use are the possibility of using a single blood sample, in addition to the speed of execution and low costs. Although in this pilot study we aimed to check the validity of the test using immunofluorescence as a downstream analysis, we stress that a further advantage is represented by the possibility of carrying out different downstream analyses on the two filters obtained from the same patient, without having to repeat the blood sampling. Further studies including a larger patient cohort and different cancer types are currently ongoing in order to validate these results.

## Figures and Tables

**Table 1:** Patient characteristics.

Characteristics	No. (%)
Age (in years)	
Mean	67.7
Range	55–84
PS	
0	4 (57)
1	3 (43)
Sex	
Male	3 (43)
Female	4 (57)
Colorectal cancer stage	
IV	7 (100)
Right-sided	4 (57)
Left-sided	3 (43)
Mutations	
RAS	4 (57)
BRAF	1 (14)

PS: performance status.

**Table 2:** CTCs and CTC clusters detection through the sequential filtration.

Patient	CTC				CTC Cluster			
	*N*_T_	CK20 (*N*)	HIF-1α (*N*)	VIM (*N*)	*N*_T_	CK20 (*N*)	HIF-1α (*N*)	VIM (*N*)
14AA6844	7	6	2	2	5	1	4	4
14AA6865	9	7	3	4	3	1	3	3
14AA6922	6	5	2	3	4	1	4	3
15AA0421	3	3	1	1	6	2	5	4
15AA0433	8	7	3	3	6	2	6	6
15AA0814	5	4	1	2	3	1	2	2
15AA0924	4	3	1	2	4	1	2	2

CTC: circulating tumor cell; CK: cytokeratin; HIF: hypoxia-inducible factor; VIM: vimentin; *N*: number; T: total.

## Data Availability

The data presented in this study are available on request from the corresponding author.

## Supplementary Materials

The following are available online at https://www.mdpi.com/article/10.3390/cancers13246362/s1, Figure S1: Hematopoietic cells identification. Representative confocal images showing CD45 positive cells observed at differential interference contrast (DIC) stained with diaminobenzidine. Magnification 60×, 5× zoom bar 10 μm. Figure S2: Illustrative images of triple immunofluorescence assay on circulating colon cancer cells clusters. Representative confocal images of CTC clusters stained with anti-CK20 (red), anti-vimentin (green) and anti-HIF-1α (yellow) antibodies. Magnification 60×, 5× zoom bar 10 μm.
