# Supplementary material for: Sequential Isolation and Characterization of Single CTCs and Large CTC Clusters in Metastatic Colorectal Cancer Patients
Source: Cancers (Basel). 2021 Dec 18;13(24):6362. doi: 10.3390/cancers13246362 (PMC8699456; doi:10.3390/cancers13246362)
Supplement: Supplementary file 1 [file cancers-13-06362-s001.zip › Supplementary Figure S1.pdf]

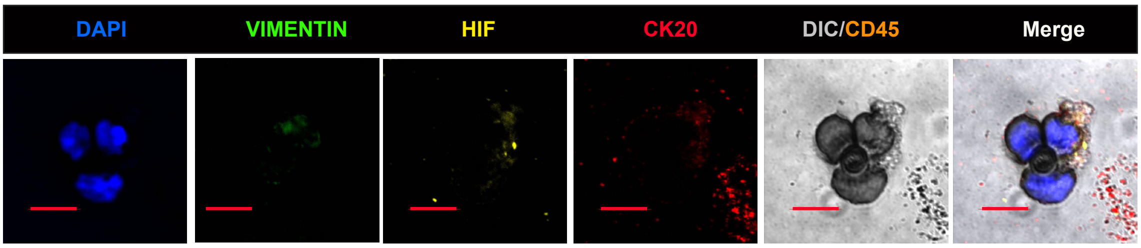

Hematopoietic cells identification. Representative confocal images showing CD45 positive cells observed at differential interference contrast (DIC) stained with diaminobenzidine. Magnification 60x, 5x zoom bar 10 μm.

Supplementary Figure S1
