# Supplementary material for: Sequential Isolation and Characterization of Single CTCs and Large CTC Clusters in Metastatic Colorectal Cancer Patients
Source: Cancers (Basel). 2021 Dec 18;13(24):6362. doi: 10.3390/cancers13246362 (PMC8699456; doi:10.3390/cancers13246362)
Supplement: Supplementary file 1 [file cancers-13-06362-s001.zip › Supplementary Figure S2.pdf]

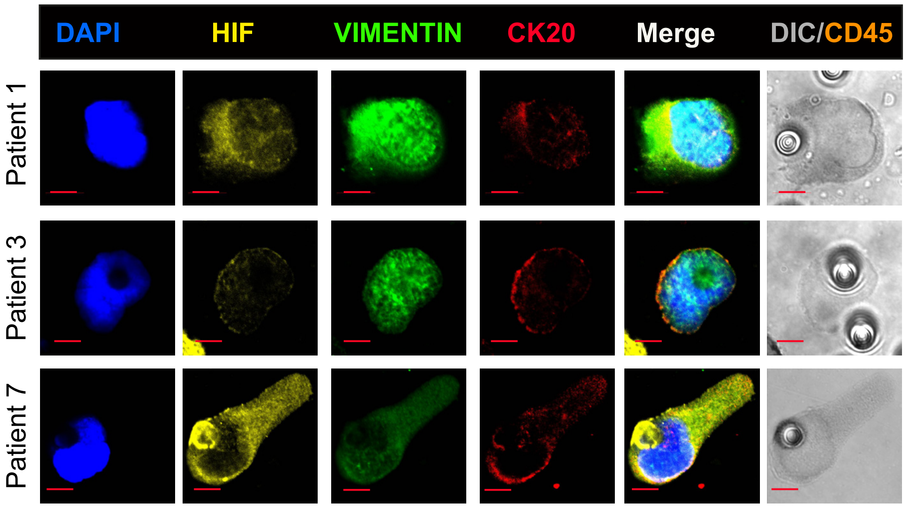

Illustrative images of triple immunofluorescence assay on circulating colon cancer cells clusters. (Left) Representative confocal images of CTC clusters stained with anti-CK20 (red), anti-vimentin (green) and anti-HIF-1α (yellow) antibodies. Magnification 60x, 5x zoom bar 10 µm.

Supplementary Figure S2
